# Cullin3 (CUL3) suppresses proliferation, migration and phenotypic transformation of PDGF-BB-stimulated vascular smooth muscle cells and mitigates inflammatory response by repressing Hedgehog signaling pathway

**DOI:** 10.1080/21655979.2021.1995572

**Published:** 2021-12-02

**Authors:** Yuluan Xiang, Lihua Li, Shuang Xia, Jinlin Lv, Xiaoling Li

**Affiliations:** a Department of Gerontology and Special Medical Services, The First Affiliated Hospital of Dali University, Dali, Yunnan, China; b Guangdong Cardiovascular Institute, Guangdong General Hospital, Guangdong Academy of Medical Sciences, Guangzhou, Guangdong, China; c Department of Cardiovascular Medicine, People’s Hospital of Fengjie, Chongqing, China

**Keywords:** Cullin3, VSMCs, Hedgehog pathway, atherosclerosis

## Abstract

Vascular smooth muscle cell (VSMC) hyperplasia is closely associated with AS progression. Hence, it is of great significance to elucidate the molecular mechanisms underlying the involvement of VSMCs in AS. SHH antagonist can inhibit the excessive proliferation, migration and phenotypic transformation of PDGF-BB-induced VSMCs. It has been proved that CUL3 can suppress Hedgehog signaling. This current work was designed to identify the biological role of CUL3 in the behaviors of VSMCs in AS and investigate the potential molecular mechanism. VSMCs were treated with PDGF-BB to establish the cell model *in vitro*. Levels of CUL3, SHH and Gli1 in PDGF-BB-stimulated VSMCs were measured by RT-qPCR analysis. Then, the precise functions of CUL3 in VSMCs were determined from the perspectives of proliferation, migration, apoptosis and phenotype transformation. Besides, the influence of CUL3 on inflammatory response in VSMCs was evaluated. Moreover, the impact of CUL3 on Hedgehog signaling pathway was also investigated. In the present research, it was observed that CUL3 was lowly expressed and SHH and Gli1 were highly expressed in PDGF-BB-stimulated VSMCs. Upregulation of CUL3 suppressed the excessive proliferation, migration and phenotypic transformation and facilitated the apoptosis of PDGF-BB-stimulated VSMCs. In addition, elevation of CUL3 alleviated inflammatory response in PDGF-BB-stimulated VSMCs. Importantly, CUL3 overexpression inactivated Hedgehog signaling pathway. To conclude, CUL3 might regulate the biological behaviors of VSMCs in AS by modulating Hedgehog signaling pathway. These data encourage to further investigate any potential therapeutic role of CUL3 in animal models of AS and explore therapeutic values for AS clinically.

## Introduction

Atherosclerosis (AS) is one kind of chronic inflammatory reactions caused by combined action of multiple factors [1]. With the acceleration of aging process in human society, the incidence rate of AS has been increasing year by year [2]. Besides, AS is the major contributor to death of cardiovascular disease worldwide, and also one of the main causes of acute cardiovascular disease [3]. This phenomenon has been widely concerned by the cardiovascular community.

AS is characterized by the deposition of lipids in the blood vessel wall, involving in inflammatory and proliferative cascade of major functional cells [4]. Vascular smooth muscle cells (VSMCs) are integral cells of the blood vessel wall, which are responsible for the relaxation and contraction of the blood vessel and responding to hemodynamic and environmental signals [5]. More and more researches have indicated that the abnormal proliferation of VSMCs plays an important role in the development of AS [6,7]. Thus, it is in urgent need to understand the molecular mechanisms underlying the involvement of VSMCs in AS, which is critical to the development of therapies for various atherosclerotic cardiovascular diseases.

Hedgehog signaling pathway was first found in the embryonic development of Drosophila melanogaster [8]. It is a highly conserved signaling pathway, which plays important roles in cell proliferation, adhesion, migration and differentiation [9,10]. Sonic hedgehog (SHH) is the most widely expressed in the Hedgehog family [11]. Besides, Gli1 is a positive regulator of Hedgehog signaling pathway [12]. The aberrant activation of Hedgehog signaling pathway is closely linked to the abnormal biological behaviors of VSMCs in AS. SHH has been confirmed to be activated in platelet-derived growth factor-BB (PDGF-BB)-induced VSMCs [13,14]. Additionally, SHH antagonist treatment could inhibit the excessive proliferation, migration and phenotypic transformation of PDGF-BB-induced VSMCs [14].

Cullin-RING E3 ligases (CRLs) are the major components of ubiquitin-proteasome system and responsible for ubiquitylation and subsequent degradation of cellular proteins [15]. Cullin3 (CUL3), a member of CRLs family, exerts an irreplaceable function in many biological processes through mediation of ubiquitination of target proteins [16]. It has been proved that CUL3 could inhibit hedgehog signaling by downregulating the expression level of the terminal transcription factor cubitus interruptus (Ci, Ci, homologous to Gli in vertebrates) [17]. However, the biological functions of CUL3 and underlying molecular mechanisms in the behaviors of VSMCs have not yet been elaborated. .

Herein, this current work was performed to identify the biological role of CUL3 in the behaviors of VSMCs in AS. The influences of CUL3 on the proliferation, migration, apoptosis and phenotypic transformation of PDGF-BB-induced VSMCs and inflammatory response were analyzed through a series of functional experiments. In addition, the impact of CUL3 on Hedgehog signaling pathway was also investigated to explore the potential molecular mechanism.

## Materials and methods

### Cell culture, transfection and treatment

VSMCs were obtained from the American Type Culture Collection (ATCC, VA, USA) and incubated in Dulbecco’s modified Eagle’s medium (DMEM, Gibco, MD, USA) with 10% fetal bovine serum (FBS; Gibco, MD, USA) and 1% penicillin/streptomycin (Gibco, MD, USA) in a humidified incubator containing 5% CO_2_ at 37°C.

VSMCs were transfected with CUL3 overexpression and interference vectors (GenePharma, Shanghai, China) using Lipofectamine 2000 (Invitrogen, MA, USA) according to the manufacturer’s instructions.

VSMCs were treated with platelet-derived growth factor (PDGF)-BB (10 ng/mL; Peprotech, NJ, USA) for 24 h.

### Cell viability assay

VSMCs (5 × 10^3^ cells/well) were seeded into 96-well plates. Post 24 h incubation, 10 μl CCK-8 reagent (Beyotime, Shanghai, China) was added into each well for additional 2 h culture. Absorbance at 450 nm was measured by a microplate reader (Bio-Tek, GA, USA).

### EdU staining assay

VSMCs were fixed with 4% paraformaldehyde for 30 min at room temperature, and then permeabilized with 0.3% TritonX-100 for 10 min. Next, VSMCs were treated with EdU reagent for 30 min in the dark. Nuclei were stained with 4′, 6-Diamidino-2-phenylindole (DAPI; Solarbio, Beijing, China) for 10 min. Stained cells were visualized under a fluorescence microscope (Leica, Wetzlar, Germany).

### Wound healing assay

Briefly, VSMCs were seeded into 12-well plates. When cells reach 90% confluence, a wound was drawn across the plate with a 200 μl pipette tip. VSMCs were then cultured in serum-free medium for 24 h. The migration distance was imaged at 0 and 24 h under an inverted microscope (Leica, Wetzlar, Germany).

### Flow cytometry

In brief, the collected VSMCs were washed with PBS and fixed in 70% ethanol for 2 h at 4°C. Next, 500 μl PI solution (Beyotime, Shanghai, China) was applied to stain VSMCs in the dark for 30 min. Then, flow cytometry was conducted using a BD flow cytometer (BD Biosciences, CA, USA).

VSMCs were resuspended with 200 µl binding buffer and stained with Annexin V-FITC and PI in the dark for 15 min using Annexin V-FITC/PI cell apoptosis staining kit (Beyotime, Shanghai, China). Then, flow cytometry was conducted using a BD flow cytometer (BD Biosciences, CA, USA).

### Western blot assay

The extraction of total proteins from VSMCs was performed by lysing cells with RIPA buffer (Beyotime, Shanghai, China), and the protein concentration was evaluated by BCA Protein Assay Kit. Then, protein samples were separated by SDS-PAGE and subsequently transferred to PVDF membranes (Millipore, MA, USA). After being blocked in 5% BSA at room temperature for 1 h, membranes were incubated overnight at 4°C with primary antibodies against α-SMA (Abcam, ab5694, 1:1000), SM22α (Abcam, ab14106, 1:1000), Bax (Abcam, ab53154, 1:1000), Bcl-2 (Abcam, ab196495, 1:2000), Caspase-3 (Abcam, ab13847, 1:500), Caspase-8 (Abcam, ab108333, 1:5000), Caspase-9 (Abcam, ab32539, 1:5000), CycD1 (Abcam, ab134175, 1:10,000), CDK4 (Abcam, ab199728, 1:2000), SHH (Abcam, ab53281, 1:5000), Gli1 (Abcam, ab134906, 1:1000) and GAPDH (Abcam, ab181602, 1:10,000). On the second day, HRP-conjugated secondary antibodies (Abcam, ab205718, 1:50,000) were employed to incubate the membranes for 1 h at room temperature. Protein bands were visualized and imaged using enhanced chemiluminescence method.

### Reverse transcription-quantitative polymerase chain reaction (RT-qPCR)

The extraction of total RNA was performed using TRIzol reagent (Invitrogen, CA, USA). cDNA was synthesized through RNA reverse transcription by PrimeScript^TM^ RT Reagent Kit (Takara, Tokyo, Japan). Then, RT-qPCR was conducted using a SYBR Premix Ex Taq^TM^ II (Takara, Tokyo, Japan). The PCR conditions were as follows: 95 °C for 10 min, followed by 40 cycles of 95 °C for 15 sec and 60 °C for 1 min. The sequences of the primers were as follows: CUL3 forward: 5′- GGAAGGAAAACAGGGAAGGTG −3′, reverse: 5′- ACATAGGAAAGGCACACAAAGGA −3′; SHH forward: 5′- CCAAGGCACATATCCACTGCT −3′, reverse: 5′- GTCTCGATCACGTAGAAGACCT −3′; Gli1 forward: 5ʹ- AGCGTGAGCCTGAATCTGTG −3ʹ, reverse 5ʹ- CAGCATGTACTGGGCTTTGAA −3ʹ; GAPDH 5′- GAAGGTGAAGGTCGGAGTC −3′, reverse: 5′- GAAGATGGTGATGGGATTTC −3′. GAPDH served as the internal control. Relative gene expressions of CUL3, SHH and Gli1 were analyzed using 2^−ΔΔCt^ method [18].

### Statistical analysis

Quantitative data were obtained from three independent experiments and expressed as mean ± standard deviation (SD). Statistical differences between two groups were determined using unpaired Student’s t-test and statistical analysis among multiple groups was analyzed by one-way analysis of variance (ANOVA) followed by Tukey’s post hoc test. Differences with a p value less than 0.05 were considered statistically significant.

## Results

### Reduced CUL3 and elevated SHH and Gli1 in PDGF-BB-stimulated VSMCs

**Figure 1.**
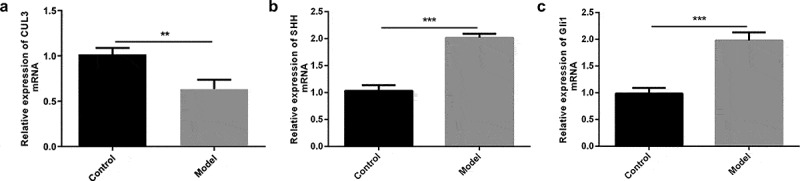
Reduced CUL3 and elevated SHH and Gli1 in PDGF-BB-stimulated VSMCs. (a-c) The mRNA expression levels of CUL3, SHH and Gli1 in PDGF-BB-stimulated VSMCs were measured by RT-qPCR assay. **P < 0.01, *** P < 0.001

To explore the biological role of CUL3 and the interaction between CUL3 and Hedgehog signaling pathway in AS, PDGF-BB was used to stimulate VSMCs as the cell model *in vitro*. CUL3 level was reduced, while SHH and Gli1 levels were elevated in VSMCs following the stimulation of PDGF-BB, suggesting that CUL3 and Hedgehog signaling pathway might participate in the development of AS (Figure 1(a-c)).

### CUL3 exerted suppressive effects on the excessive proliferation of PDGF-BB-stimulated VSMCs

**Figure 2.**
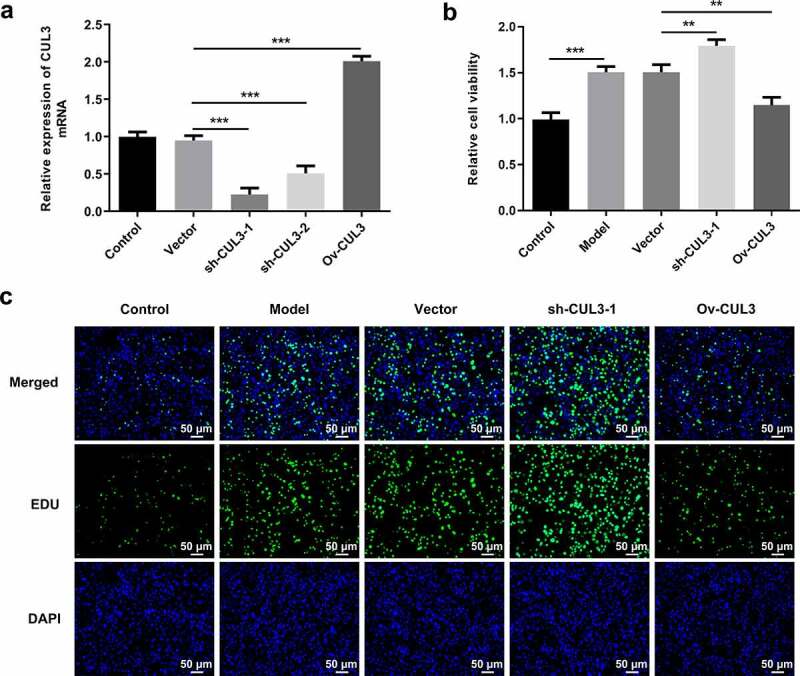
CUL3 exerted suppressive effects on the excessive proliferation of PDGF-BB-stimulated VSMCs. Cells were transfected with sh-CUL3 or Ov-CUL3. (a) The transfection efficiency was validated by RT-qPCR analysis. (b) Viability of VSMCs was measured by CCK-8 assay. (c) VSMCs proliferation was evaluated by EdU staining. **P < 0.01, *** P < 0.001

**Figure 3.**
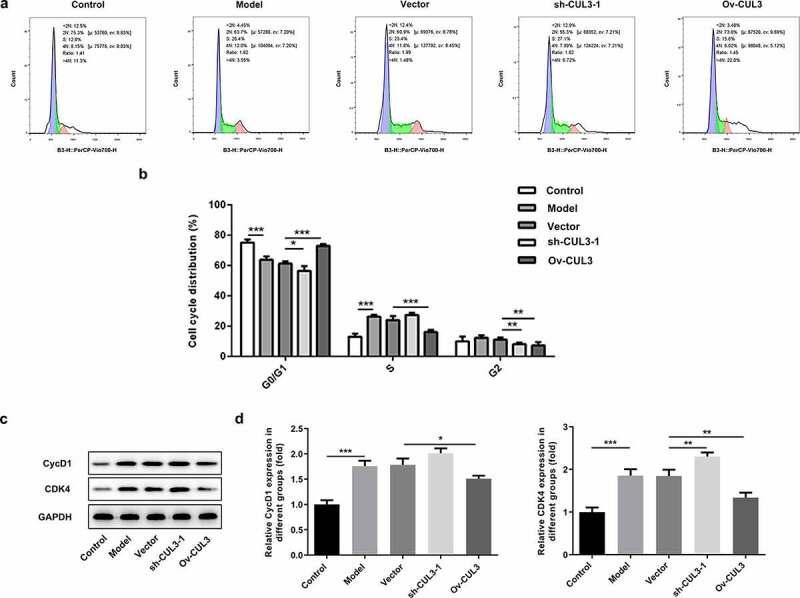
CUL3 exerted suppressive effects on the excessive proliferation of PDGF-BB-stimulated VSMCs. Cells were transfected with sh-CUL3 or Ov-CUL3. (a, b) Cell cycle distribution was analyzed by flow cytometry analysis. (c, d) The protein expression levels of CycD1 and CDK4 were detected by western blot assay. *P < 0.05, **P < 0.01, *** P < 0.001

In order to identify the precise functions of CUL3 in the biological behaviors of VSMCs, sh-CUL3-1, sh-CUL3-2 or Ov-CUL3 were introduced to downregulate or upregulate CUL3 expression, and the transfection efficiency was validated by RT-qPCR analysis (Figure 2(a)). PDGF-BB stimulation induced excessive VSMCs proliferation. Results of CCK-8 assay indicated that downregulation of CUL3 significantly promoted the proliferation of PDGF-BB-induced VSMCs and upregulation of CUL3 exerted the opposite effects on VSMCs proliferation (Figure 2(b)). Moreover, EdU staining further verified that CUL3 exerted suppressive effects on the excessive proliferation of PDGF-BB-stimulated VSMCs (Figure 2(c)). In addition, flow cytometry analysis of cell cycle distribution revealed that transfection with Ov-CUL3 increased the percentage of cells in G0/G1 phase. Meanwhile, the percentage of cells in S and G2 phases was decreased in a certain extent. Upregulation of CUL3 visibly induced cell cycle arrest of VSMCs, inhibiting the excessive proliferation of PDGF-BB-stimulated VSMCs. Downregulation of CUL3 showed the opposite effect (Figure 3(a, b)). Besides, elevated expressions of CycD1 and CDK4 caused by CUL3 knockdown and reduced expressions of CycD1 and CDK4 caused by CUL3 overexpression evidenced that CUL3 boosted cell cycle arrest of VSMCs to suppress the excessive proliferation of PDGF-BB-stimulated VSMCs (Figure 3(c, d)).

### CUL3 exerted suppressive effects on the excessive migration of PDGF-BB-stimulated VSMCs

**Figure 4.**
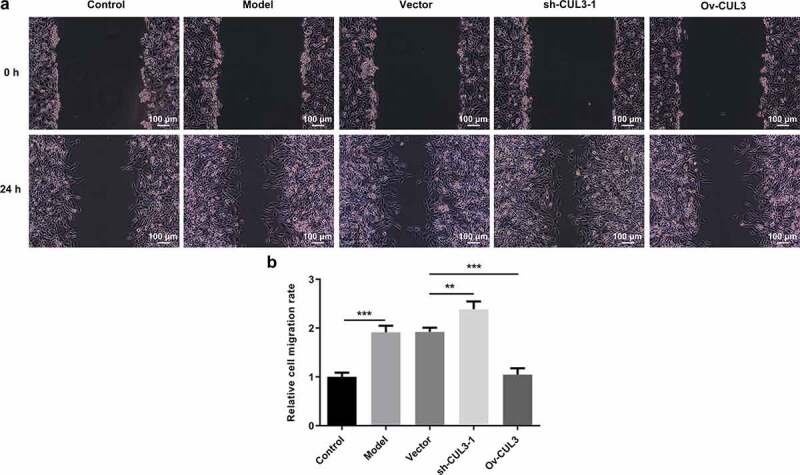
CUL3 exerted suppressive effects on the excessive migration of PDGF-BB-stimulated VSMCs. Cells were transfected with sh-CUL3 or Ov-CUL3. (a, b) VSMCs migration was evaluated by wound healing assay. **P < 0.01, *** P < 0.001

Furthermore, migration capabilities of VSMCs were evaluated via wound healing assay. PDGF-BB stimulation induced excessive VSMCs migration. Then, it was observed that downregulation of CUL3 promoted the migration of PDGF-BB-stimulated VSMCs and upregulation of CUL3 repressed the migration of PDGF-BB-stimulated VSMCs (Figure 4(a, b)).

### CUL3 facilitated the apoptosis of PDGF-BB-stimulated VSMCs

**Figure 5.**
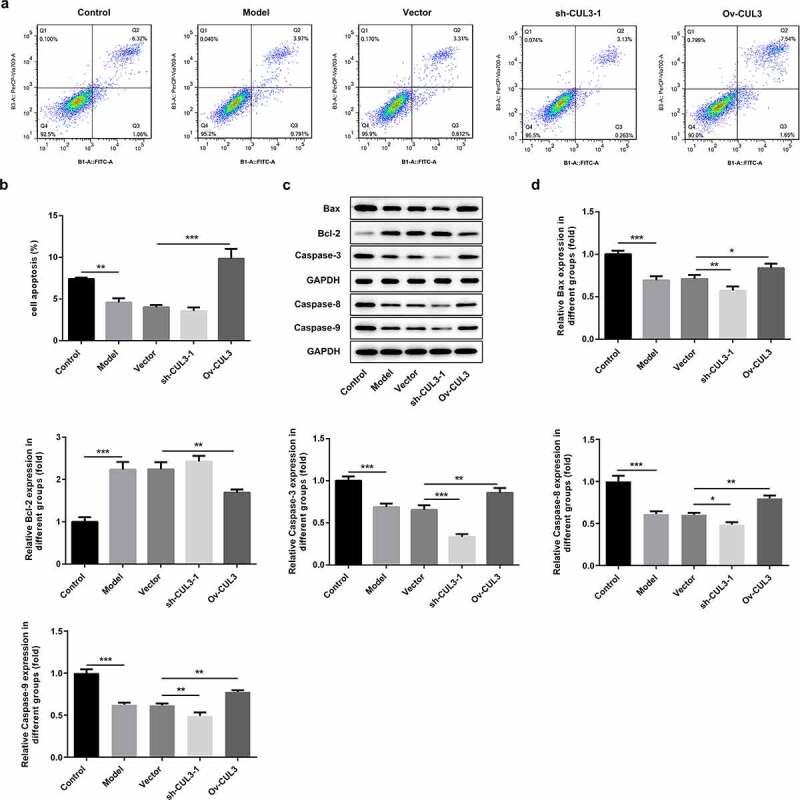
CUL3 facilitated the apoptosis of PDGF-BB-stimulated VSMCs. Cells were transfected with sh-CUL3 or Ov-CUL3. (a, b) VSMCs apoptosis was measured by flow cytometry analysis. (c, d) The protein expression levels of Bax, Bcl-2, Caspase-3, Caspase-8 and Caspase-9 were detected by western blot assay. *P < 0.05, **P < 0.01, *** P < 0.001

Additionally, the influence of CUL3 on VSMCs apoptosis was also investigated. PDGF-BB stimulation repressed VSMCs apoptosis. Results of flow cytometry analysis suggested that downregulation of CUL3 further inhibited VSMCs apoptosis and upregulation of CUL3 facilitated the apoptosis of PDGF-BB-stimulated VSMCs (Figure 5(a, b)). Besides, decreased expressions of Bax, Caspase-3, Caspase-8 and Caspase-9 and increased expression of Bcl-2 following transfection with sh-CUL3 suggested that downregulation of CUL3 further inhibited the apoptosis of PDGF-BB-stimulated VSMCs. In contrast, elevated expressions of Bax, Caspase-3, Caspase-8 and Caspase-9 and reduced expression of Bcl-2 following transfection with Ov-CUL3 indicated that upregulation of CUL3 facilitated the apoptosis of PDGF-BB-stimulated VSMCs (Figure 5(c, d)).

### CUL3 repressed the phenotypic transformation of PDGF-BB-stimulated VSMCs

**Figure 6.**
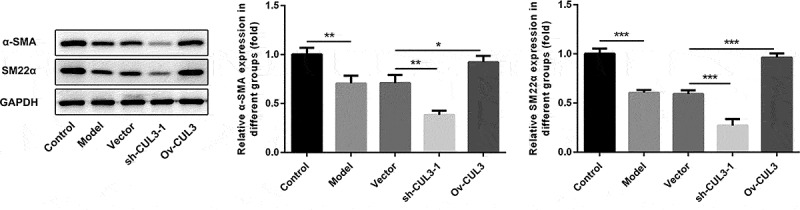
CUL3 repressed the phenotypic transformation of PDGF-BB-stimulated VSMCs. Cells were transfected with sh-CUL3 or Ov-CUL3. The protein expression levels of α-SMA and SM22α were detected by western blot assay. *P < 0.05, **P < 0.01, *** P < 0.001

PDGF-BB stimulation boosted VSMCs phenotypic transformation. Moreover, it was observed that CUL3 knockdown decreased α-SMA and SM22α expression in VSMCs under PDGF-BB stimulation, while CUL3 overexpression increased α-SMA and SM22α expression in PDGF-BB-stimulated VSMCs. These results suggested that CUL3 inhibited VSMCs phenotypic transformation (Figure 6).

### CUL3 relieved inflammatory response in PDGF-BB-stimulated VSMCs

**Figure 7.**
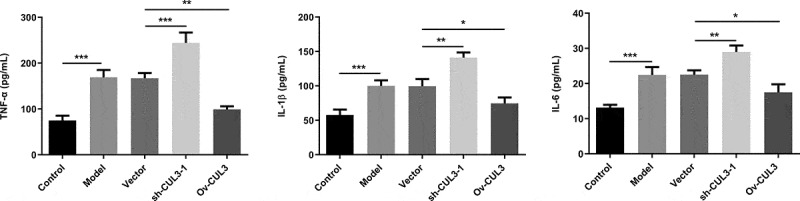
CUL3 relieved inflammatory response in PDGF-BB-stimulated VSMCs. Cells were transfected with sh-CUL3 or Ov-CUL3.Levels of TNF-α, IL-β and IL-6 were respectively assessed using ELISA kits. *P < 0.05, **P < 0.01, *** P < 0.001

Obvious inflammatory response was observed in VSMCs under the stimulation of PDGF-BB. Levels of TNF-α, IL-β and IL-6 were further increased following downregulation of CUL3 in comparison with those in PDGF-BB-stimulated VSMCs. In addition, CUL3 overexpression significantly reduced the release of these pro-inflammatory cytokines (Figure 7). These experimental findings indicated that CUL3 mitigated obvious inflammation in VSMCs stimulated by PDGF-BB.

### CUL3 functioned via regulating Hedgehog signaling pathway

**Figure 8.**
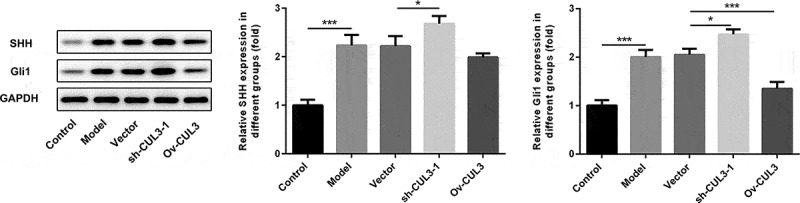
CUL3 functioned by regulating Hedgehog signaling pathway in PDGF-BB-stimulated VSMCs. Cells were transfected with sh-CUL3 or Ov-CUL3. The protein expression levels of SHH and Gli1 were detected by western blot assay. *P < 0.05, *** P < 0.001

To explore the underlying molecular mechanism, the relationship between CUL3 and Hedgehog signaling pathway in VSMCs was then confirmed. PDGF-BB stimulation activated Hedgehog signaling pathway in VSMCs. Downregulation of CUL3 elevated the protein levels of SHH and Gli1, whereas upregulation of CUL3 reduced the protein levels of SHH and Gli1 in PDGF-BB-stimulated VSMCs. These findings suggested that CUL3 might regulate the biological behaviors of VSMCs in AS by modulating Hedgehog signaling pathway (Figure 8).

## Discussion

AS has been recognized as a momentous pathological basis for cardiovascular diseases. The pathogenesis of AS is incredibly complex and has not yet been fully elucidated [1,3]. The biological behaviors of VSMCs exert important effects on the pathogenesis and development of AS. AS is probably caused by pathophysiological changes in VSMCs such as hyperproliferation, hypertrophy, migration and inflammation [19].

CRLs are a big family of ubiquitin E3 ligases composed of similar architectures, including a scaffold Cullin protein, a Ring protein and a substrate receptor module. Different Cullin proteins decide the presence of other components in the same CRL complex [20,21]. The dysregulation of CUL3 shares a strong association with the progression of AS. CUL3 could mediate ABC subfamily A member 1 (ABCA1) ubiquitination and degradation to inhibit cholesterol efflux and contribute to the pathogenesis of AS [22]. In the present research, it was confirmed that CUL3 was obviously reduced in PDGF-BB-stimulated VSMCs. PDGF-BB stimulation induced excessive proliferation and migration of VSMCs, repressed apoptosis of VSMCs as well as activated inflammatory response. CUL3 exerted suppressive effects on the excessive proliferation and migration of PDGF-BB-stimulated VSMCs, facilitated the apoptosis of PDGF-BB-stimulated VSMCs as well as relieved inflammation.

Structural and functional changes of VSMCs are closely correlated with the occurrence and development of AS, may promote the plaque formation and increase the instability of plaque [23]. In the present study, VSMCs phenotypic transformation was evaluated. PDGF-BB stimulation boosted VSMCs phenotypic transformation, while CUL3 repressed the phenotypic transformation of PDGF-BB-stimulated VSMCs. Therefore, it was speculated that CUL3 could participate in the progression of AS by regulating the structure and biological functions of VSMCs.

Hedgehog signaling pathway includes secretory glycoprotein ligand Hedgehog, transmembrane protein receptor Patched, transmembrane protein Smoothened, nuclear transcription factor Gli protein, suppress of Fu (SuFu) and downstream target gene [24]. Zeng et al. have revealed that activation of SHH/Gli2 signaling could enhance the effect of PDGF in inducing VSMCs dedifferentiation, while inhibition of SHH could reduce VSMCs dedifferentiation [13]. Besides, Xu et al. [14] have also proved that SHH is activated in PDGF-BB-induced VSMCs, and activation of SHH could exacerbate the proliferation, migration and phenotypic transformation of PDGF-BB-induced VSMCs. In our work, elevated expressions of SHH and Gli1 were observed in PDGF-BB-stimulated VSMCs. A previous study has pointed out that CUL3 could induce Gli1 ubiquitination degradation and inhibit Hedgehog signaling [25]. Results of this current work also suggested that upregulation of CUL3 repressed Hedgehog signaling pathway in PDGF-BB-stimulated VSMCs. These experimental findings prompted that Hedgehog signaling pathway may play vital roles in the pathogenesis of AS.

## Conclusion

To sum up, this study validated that CUL3 was lowly expressed in PDGF-BB-stimulated VSMCs. Functional experiments verified that CUL3 suppressed the excessive proliferation, migration and phenotypic transformation of PDGF-BB-stimulated VSMCs, facilitated the apoptosis of PDGF-BB-stimulated VSMCs and alleviated inflammation by inactivating Hedgehog signaling pathway.

## Data Availability

The analyzed data sets generated during the present study are available from the corresponding author on reasonable request.
